# An Electrically Rechargeable Zinc/Air Cell with an Aqueous Choline Acetate Electrolyte

**DOI:** 10.3390/ma13132975

**Published:** 2020-07-03

**Authors:** Mariappan Sakthivel, Sai Praneet Batchu, Abbas Ali Shah, Kwangmin Kim, Willi Peters, Jean-Francois Drillet

**Affiliations:** DECHEMA-Forschungsinstitut, Theodor-Heuss-Allee 25, 60486 Frankfurt am Main, Germany; sakthivel@dechema.de (M.S.); bspraneet@gmail.com (S.P.B.); abbasalishah@live.com (A.A.S.); kwangmin.m.kim@gmail.com (K.K.); peters@dechema.de (W.P.)

**Keywords:** zinc/air battery, choline acetate, Bi-functional spinel catalyst, Oxygen reduction reaction (ORR) – oxygen evaluation reaction (OER) activity

## Abstract

Due to the feasibility of an electrically rechargeable zinc/air cell made of a zinc foil as active material, an aqueous choline acetate (ChAcO) mixture as an electrolyte and a spinel MnCo_2_O_4_ (MCO) and NiCo_2_O_4_ (NCO) as a bi-functional oxygen catalyst was investigated in this work. The 30 wt.% water-containing aqueous ChAcO solution showed high contact angles close to those of KOH favoring triple-phase boundary formation in the gas diffusion electrode. Conductivity and pH value of 30 wt.% H_2_O/ChAcO amounted to 5.9 mS cm^−1^ and 10.8, respectively. Best results in terms of reversible capacity and longevity of zinc/air cell were yielded during 100 h charge/discharge with the MnCo_2_O_4_ (MCO) air electrode polarization procedure at 100 µA cm^−2^ (2.8 mA g^−1^_zinc_). The corresponding reversible capacity amounted to 25.4 mAh (28% depth of discharge (DOD)) and the energy efficiency ranged from 29–54% during the first and seventh cycle within a 1500 h polarization period. Maximum active material utilization of zinc foil at 100 µA cm^−2^ was determined to 38.1 mAh (42% DOD) whereas a further charging step was not possible due to irreversible passivation of the zinc foil surface. A special side-by-side optical cell was used to identify reaction products of the zinc/air system during a single discharge step in aqueous ChAcO that were identified as Zn(OH)_2_ and ZnO by Raman analysis while no carbonate was detected.

## 1. Introduction

The demand for efficient, low-cost, and energy storage systems for portable, mobile, and stationary applications is increasing dramatically. Currently, a traditional system such as lead-acid and Ni-Cd are dominating the market for electricity supply of back-up units, a pallets transporter, energy storage of solar collectors, and brake energy recuperation in trains. Especially in Germany where old nuclear plants will be successively replaced by a biomass/wind/photovoltaic mix, new battery technologies are required for storing surplus electricity produced during windy and/or sunny days and by the way buffer acts for seasonal variations. In past decades, many efforts aimed to develop alkaline zinc/air battery whose theoretical capacity amounts to 820 mAh g^−1^_zinc_. Battery compounds are relatively cheap, non-toxic, and not prone to thermal runaway reactions as in the case of Li-ion [1]. However, with the exception of the primary zinc/air battery for hearing aids devices, wide introduction of electrically rechargeable systems into the consumer market is hindered by numerous inherent technical drawbacks such as passive layer formation and dendrite growing at the metal electrode [2], electrolyte evaporation and contamination, pore plugging at the gas diffusion electrode (GDE) due to flooding and carbonate formation [3], and insufficient kinetics and stability of the oxygen catalyst for both oxygen reduction (ORR) and evolution (OER) reactions that affect overall energy efficiency (<60%). In Zn-air cell with alkaline electrolyte, the electrode reaction and, during the discharge process and corresponding theoretical potential values, are shown below.
Zinc electrode: Zn + 4OH^−^ → [Zn(OH)_4_]^2^^−^ + 2e^−^(E_0_ = −1.26 V)[Zn(OH)_4_]^2−^ → ZnO + H_2_O + 2OH^−^
Air electrode: O_2_ + 2H_2_O + 4e^−^ → 4OH^−^(E_0_ = +0.4 V)Overall reaction: 2Zn + O_2_ → 2ZnO(E_0_ = +1.66 V)

During the charging step, reverse reactions occur.

While deficits linked to zinc electrode and electrolyte appear to be technically solvable, problems related to the air electrode are rather inherent to thermodynamic issues of ORR (strong O=O bond) and OER reactions including hydrogen peroxide side reaction and poor chemical stability in a highly alkaline environment. In order to increase both activity and stability of the reversible air cathode, there are three main strategies that rely on: (i) mixture of ORR and OER catalysts (e.g., Ag + W_2_C) [4], (ii) hybrid bi-electrodes (e.g., MnO_2_/NiCo) [5], and (iii) bi-functional catalysts (e.g., La_0.6_Ca_0.4_CoO_3_ (LCCO) perovskite). According to Sintivich et al. [6], the transition-metal oxide in perovskite should have a surface-cation e_g_ occupancy close to unity and high B-site oxygen covalency similarly to Ba_0.5_Sr_0.5_Co_0.8_Fe_0.2_O_3_ (BSCF) structure for yielding high OER activity. Bogolowski et al. reported higher activity of BSCF/C_65_ over 3000 h at 10 mA cm^−^^2^ for both OER and ORR in comparison with LCCO/C_65_ GDE [7].

Metal oxides with a spinel structure are promising bifunctional oxygen catalysts as well [8]. Pletcher et al. reported on Ni foam-based GDE with a NiCo_2_O_4_/ Polytetrafluorethylen (PTFE) active layer that was able to deliver high current densities up to 100 mA cm^−2^ [9]. Gebremariam et al. investigated a Zn/air battery based on manganese cobalt oxide nanoparticles anchored on carbon nanofibers and wrapped in a nitrogen-doped carbon shell (MnCo_2_O_4_/CNFs@NC) catalyst [10]. They yielded a specific capacity of 695 mAh g^−1^_Zn_ and an energy density of 778 Wh kg^−1^_Zn_ at a current density of 20 mA cm^−2^. Some other Co-based systems such as CoMn_2_O_4_/N-rGO [11], MnCo mixed oxide [12], and Co(II)_1−x_Co(0)_x_/3Mn(III)_2x_/3S nanoparticles supported on B/N-co-doped mesoporous nanocarbon NiCo_2_O_4_ [13,14,15], NiCo_2_O_4_/NCNT [16], and CoSx@N or S co-doped graphene nanosheets [17] were also tested in the zinc/air cell. Ishihara et al. developed a mesoporous MnCo_2_O_4_ with a high surface area of 108 m^2^ g^−1^ that substantially lowered ORR/OER overpotential in 4 M KOH and allowed high reversible capacity values that are more than 250 cycles with a zinc foil [18]. Wang et al. demonstrated high throughput production of Mn (IV) and Co (II)-rich surface MnCo_2_O_4_ bifunctional electrode (low ΔE = 0.83 V) by an ultrasonic humidifier aerosol-route at 480 °C [19].

To counterpart battery dry-out, substitution of the alkaline solution by a protic ionic liquid (IL) with low vapor pressure seems to be an appealing approach [20]. The other key advantages of customized ILs for electrochemical applications are (i) wide electrochemical potential window up to 2–5 V, (ii) high solubility of metal salts (e.g., chloride), (iii) water mix ability of protic species [21], and (iv) high conductivity compared to non-aqueous solvents (up to ~10 mS cm^−^^1^) [22]. It should be emphasised that only a minority of ILs possesses all these properties. An extensive survey of alternative electrolytes for zinc-based batteries can be consulted in reference [23]. Moreover, they have to favour both Zn and oxygen reactions and exhibit sufficient surface tension to enable triple-phase boundary formation. In a preliminary screening step, some acetate-based ILs such as EMIMAcO and choline acetate (ChAcO) showed adequate surface tension values and higher contact angle values than 110° on Pt/C + 20 wt.% PTFE GDE compared to, e.g., DEMAOTf [24]. Additionally, their good affinity to H_2_O makes them even more interesting.

**Figure 1:**
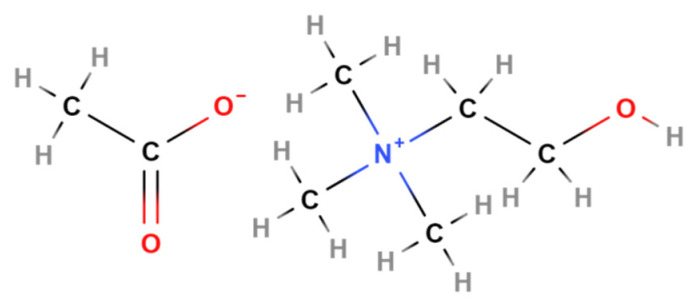
Chemical structure of 2-hydroxyethyl-trimethylammonium acetate [(C_2_H_4_OH)-(CH_3_)_3_N] [acetate] (choline acetate).

This work focuses more specifically on the test of aqueous choline acetate (2-hydroxyethyl-trimethylammonium acetate) in combination with bifunctional manganese or nickel cobalt spinel oxygen catalysts that already exhibited easy tunable catalytic properties [25] and high activity in alkaline fuel cell [26]. Choline acetate is promising because of its relatively high ionic conductivity [27], bio-degradability, low-cost production [28], and high affinity to water. Thermophysical investigations of aqueous choline acetate binary solution revealed that strong solute-solvent interactions get stronger with an increase in temperature while computational studies confirmed a decrease in solute-solute interactions with an increase in aqueous solvent concentration [29]. The chemical structure of choline acetate is shown in Figure 1.

In this work, the behavior of 30 wt.% water-containing choline acetate [(C_2_H_4_OH)-(CH_3_)_3_N] [Acetate] in terms of contact angle, activity for zinc and air electrode reactions, and its interaction with ambient air will be investigated. Furthermore, the nature of reaction products during the zinc/air cell discharge step will be identified by Raman analysis.

## 2. Materials and Methods

### 2.1. Hydrothermal Synthesis of MnCo_2_O_4_ (MCO) and NiCo_2_O_4_ (NCO)

A total of 25 mL of 50 mM manganese(II) nitrate hydrate (Mn(NO_3_)_2_∙4H_2_O, 99.99%, Sigma-Aldrich, Taufkirchen, Germany) or nickel(II) nitrate hexahydrate (Ni(NO_3_)_2_∙6H_2_O, 99.999% Sigma-Aldrich, Taufkirchen, Germany) were mixed with 25 mL of 100 mM cobalt(II) nitrate hexahydrate (Co(NO_3_)_2_∙6H_2_O, 99.999%, Sigma-Aldrich, Taufkirchen, Germany) at 250 rpm for 30 min, as reported elsewhere [25]. Subsequently, 5.2 mL of 1.25 M NaOH solution was added dropwise into the reaction mixture under vigorous stirring at 500 rpm for 30 min until the color of the solution turned brown or dark green in case of Mn(NO_3_)_2_∙4H_2_O and Ni(NO_3_)_2_∙6H_2_O precursor, respectively. Afterward, the mixture was transferred into 60 mL vials and the hydrothermal process was carried out at 160 °C for 10 h in a drying oven (FD 23, Binder, Tuttlingen, Germany). The black-colored hydroxide was collected by centrifugation (3K30, Sigma, Germany) at 10,000 rpm for 3 min, washed with deionized water until attaining a pH value of 7, and stored overnight in a freeze-drying unit (L06, Dieter Piatkowski-Forschungsgeräte, Petershausen, Germany) at −50 °C and 50 mbar. Then, the powder was grounded manually in a mortar and calcinated in a tubular oven (RHTC 80-710/15, Nabertherm, Lilienthal, Germany) at 400 °C for 1 h.

### 2.2. Fabrication of Gas Diffusion Electrode (GDE)

A 36 cm^2^ sized 20 wt.% PTFE-coated Toray carbon paper (TP-060T, QuinTech, Göppingen, Germany) was used as a gas diffusion layer (GDL). The catalyst ink was prepared by successively dispersing as-prepared powder catalyst, C_65_ carbon (C-NERGY, TIMCAL, Bironico, Switzerland), and PTFE binder (60:20:20 wt.%) in 2 mL of a 1:2 weight ratio water:isoproponal mixture, ultrasonicated for 2 min, and then sprayed on GDL. The coated electrodes were sintered at 80 °C in air between each spraying step for 3 min and for 1 h after reaching the overall catalyst loading of 1 mg cm^−2^. A disk electrode of 2.54 cm^2^ geometrical area was coined from a GDE sheet for electrochemical measurements in Plexiglas and El-Cell cells.

### 2.3. Preparation and Characterization of Aqueous Ionic Liquid Electrolyte

For ZnAcO-containing electrolyte preparation, 1.97 g ChAcO (C_7_H_17_NO_3_, 98%, IoLiTec, Heilbronn, Germany), 30 wt.% H_2_O related to choline acetate mass and 3.32 mg Zn(AcO)_2_ (99.99%, Sigma-Aldrich, Taufkirchen, Germany) were mixed in a glass vessel under ambient atmosphere and stirred for 30 min. In order to determine conductivity and pH value of different H_2_O:ChAcO mass ratios, water was added stepwise to as-received choline acetate. A large electrolyte sample of at least 20 g was used for each measurement to reduce the weighing error. Due to the highly hygroscopic nature of ChAcO, the samples were purged with argon and kept closed during pH (GMH 5340/GE117 Greisinger, Regenstauf, Germany) and conductivity (LF40 in combination with LTC0,35/23 electrode, Meinsberg, Waldheim, Germany) measurements. The solution density was determined by using a simple narrow necked volumetric flask (20 mL).

### 2.4. Structural Characterization Techniques

The X-ray diffraction patterns were collected with a diffractometer (D8 Advance, Bruker, Karlsruhe, Germany) using nickel filtered Cu K-alpha source with λ = 0.154 nm. Catalyst morphology and composition were investigated by scanning electron microscopy (SEM)/energy dispersive x-ray spectroscopy (EDX) (XL 40, Philips, Eindhoven, The Netherlands) and transmission electron microscopy (TEM) (EM 420, Philips, Eindhoven, The Netherlands). The GDE/electrolyte drop interface was studied by contact angle goniometer (OCA 15Pro, Dataphysics, Filderstadt, Germany) in ambient air at 23 °C. In order to study the reaction products during the zinc/air discharge step, a side-by-side optical cell (ECC-opto-SBS, El-Cell, Hamburg, Germany) and a confocal Raman microscope (InVia Reflex, Renishaw, Kingswood, UK) were employed. The laser beam (Ar, 532 nm, 2 mW) was focused through a 50× objective lens (DM 2500, Leica, Mannheim, Germany) with ~1 mm spot size. A high-resolution diffraction grating density of 1800 grooves mm^−^^1^ was employed. A single spectrum consisted of 20 accumulated scans with an acquisition time of 20 s each. The background signal was subtracted according to the baseline correction mode.

### 2.5. Half-Cell Measurements

#### 2.5.1. Catalyst Activity for ORR/OER Reactions

All half-cell experiments were carried out with a potentiostat/galvanostat (IM6eX, Zahner, Kronach, Germany). Tests of bifunctional GDE (2.54 cm^2^) for OER/ORR were performed in a Poly ether ether ketone (PEEK) cell (FlexCell, Gaskatel, Kassel, Germany) equipped with a Pt-coated Ti sheet as counter and a reversible hydrogen electrode (RHE) as a reference electrode. Two experimental protocols were applied with respect to electrolyte used in order to avoid any deterioration of the catalyst structure in 7 M KOH. Especially during a cathodic scan where molecular oxygen is produced, a maximal potential scanning rate and current density value was set to 5 mV s^−1^ and 100 mA cm^−2^, respectively. For investigation in ionic liquid, the maximum current density was fixed to 10 mA cm^−2^. All experiments were carried out with synthetic dry air (79.5% N_2_ + 20.5% O_2_, Linde GmbH, Offenbach, Germany) at 10 mbar overpressure. Residual moisture was removed from the synthetic air feed by connecting a gas drying unit (Drierite™) filled with CaSO_4_ pellets (Sigma-Aldrich, Taufkirchen, Germany). A long-term stability protocol consisted of 2.0 h for OER (charging) and ORR (discharging) step at 100 µA cm^−2^ each with a 30 min interruption at open circuit voltage (OCV) in-between.

#### 2.5.2. Zn Redox Reaction

The zinc redox reactions were investigated in an El-Cell (ECCair, El-Cell, Hamburg, Germany) laboratory cell. A zinc foil (Ø = 18 mm, 50 µm in thickness, 90 mg, 99.999%, Chempur, Karlsruhe, Germany) was used as a substrate, a Pt/C GDE (EC-20-10-7P, Ø = 18 mm, QuinTech, Göppingen, Germany) was used as a counter, a zinc wire (Ø = 1 mm, 99.999%, Chempur, Karlsruhe, Germany) was used as a pseudo-reference electrode, and a glass fiber separator (Ø = 18 mm,1.55 mm in thickness, El-Cell, Hamburg, Germany) was used as a separator. In 7 M KOH, the potential window was set between −400 and +400 mV while, in ChAcO, it was extended from −400 to +900 mV. The potential loss (IR-drop) was determined by electrochemical impedance spectroscopy at open circuit voltage (OCV) and corrected in half cell electrochemical data analysis. All potential values were normalized to the normal hydrogen electrode (NHE).

### 2.6. Full-Cell Measurements

#### 2.6.1. Current-Voltage and Charge/Discharge Characteristics in the El-Cell

The zinc/air battery test measurements were performed with the help of a battery cycler (BCS 10, Biologic Scientific Instruments/Gamec Analysentechnik, Illingen, Germany). A schematic diagram of the three-electrode El-Cell design is provided in Figure S1a. The El-Cell was assembled by successive pile-up of zinc foil, borosilicate glass fiber separator filled with 300 μL of alkaline or IL electrolyte, and GDE. The use of a zinc foil in spite of state-of-the-art zinc powder was motivated by easily handling during cell fabrication. A commercial 40 wt.% Pt/C benchmark GDE (EC-20-10-7P, QuinTech, Göppingen Germany) was tested for comparison. Before the test, the cell was purged with synthetic dry air for 6 h. The cell performance was evaluated first with the voltage/current (U/I) protocol by applying constant current densities in the range of 1–100 μA cm^−2^ for 15 min charge/discharge duration and 15 min OCV at each step (1 h/cycle) under either dry or ambient air conditions. Due to the poor conductivity of ionic liquid compared to that of alkaline electrolyte, the OCV step was introduced between charge/discharge steps in full cell experiments to observe the transition period to an equilibrium state. After initial characterization, the cell was subjected to a cyclic stability procedure at 100 μA cm^−2^ charge/discharge step for 8 h over one month (26 h/cycle) under ambient air condition (30% relative humidity (RH) at 23 °C). Lastly, best performing catalyst systems were investigated at 100 μA cm^−2^ for 100 h charge/discharge step (10 days/cycle) in an ambient air condition (30% RH at 23 °C).

#### 2.6.2. Charge/Discharge Characteristic in the Coin Cell

The coin cell (CR-2032) was fabricated by assembling a zinc foil (Ø = 16 mm, 0.05 mm thickness, 70 mg, 99.99%, Goodfellow, Friedberg, Germany), 2 glass fiber separators (Ø = 18 mm, 1 mm thickness, Whatman) filled with 250 µL of ChAcO + 30 wt.% H_2_O + 0.01 M ZnAcO electrolyte, and an as-prepared GDE (Ø = 18 mm) with a crimper (MSK-PN110-S, MTI, Richmond, VA, USA) system at an air pressure of 6 bar for 5 s. The GDE surface was ventilated through four pre-drilled holes (Ø = 1 mm) in the cover case (see Figure S1b). The polarization profile consisted of 5 h charge/discharge steps and 2 h OCV (12 h/cycle) under ambient air conditions (30% RH at 23 °C).

### 2.7. In-Situ Raman Investigation During Zn/Air Discharge Step in IL

First, a zinc electrode with a well-defined state-of-charge (SOC) was designed by electrodeposition of 15 mg zinc from ChAcO + 30 wt.% H_2_O + 0.1 M ZnAcO solution at 1 mA for 12 h (12 mAh at 100% current efficiency) on a PTFE-free gas diffusion layer (TGPH90, Toray, QuinTech, Göppingen, Germany). For in-situ Raman experiment, a commercially available side-by-side (SBS) optical cell (El-Cell) was used. Hereby, both electrodes (10 mm^2^) and the glass fibre separator (1 mm) are aligned in the same plane (see Section 3.4.). Then, 50 µL of ChAcO + 30 wt.% H_2_O solution was filled in a manner that no air bubbles arise between the glass and electrode surface. Air circulation was provided by small holes in the cell body.

## 3. Results and Discussion

### 3.1. Physicochemical Characterization of Catalyst Powder

**Figure 2:**
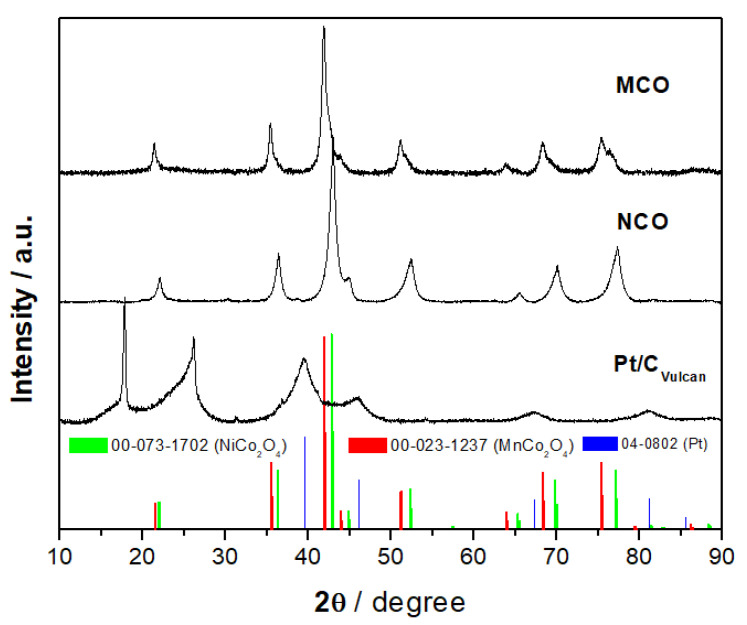
XRD patterns of as-prepared NiCo_2_O_4_ and MnCo_2_O_4_ as well as Pt/C powder from a commercial GDE.

Figure 2 shows X-ray diffraction (XRD) patterns of as-prepared MnCo_2_O_4_ (MCO) and NiCo_2_O_4_ (NCO) catalyst powder and their respective standard. The diffraction spectra of the two as-prepared powder materials match perfectly the cubic structure of AB_2_O_4_ (A = Mn, Ni and B = Co) with the Fd3m space group (JCPDS 00-023-1237 (MnCo_2_O_4_) and JCPDS 00-073-1702 (NiCo_2_O_4_), where A-site atoms are located in octahedral and B atoms occupy both octahedral and tetrahedral sites. No additional foreign peaks were detected in the XRD pattern of MCO and NCO indicating the high purity of the catalyst preparation by a hydrothermal route, which is followed by a calcination step at 400 °C. The collected XRD spectra are in good agreement with those from literature such as NCO from hydrothermal [30] and MCO from sol-gel and flame spray pyrolysis methods [31]. Since the XRD pattern of pure Ni is also located at 44.5° [JCPDS 04-0850], we cannot definitely exclude the presence of a residual Ni phase in an as-prepared NCO sample.

**Figure 3:**
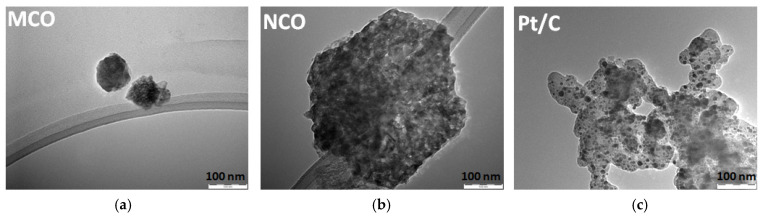
TEM images of as-prepared (**a**) MnCo_2_O_4_ (MCO), (**b**) NiCo_2_O_4_ (NCO), and (**c**) as-received Pt/C from commercial EC_20_ GDE.

Figure 3 shows TEM images of MCO and NCO powder particles synthesized by the hydrothermal route after a calcination step at 400 °C (see SEM image and EDX pattern in Figure S2). While MCO particles exhibit a relatively small spherical shape (30–50 nm) [32], NCO material is made of a rather large 2D hexagonal [33] (200 nm) flake structure that consists of smaller primary particles in the range of 8 to 26 nm. These latter are in a similar range as carbon-supported Pt ones (10 nm). BET-specific surface area and average pore size of as-synthesized spinel powders is listed in Table 1 and their respective isotherm curves and pore size distribution plots are provided in Figure S3. Especially, the BET value of MCO (120 m^2^ g^−1^) is substantially higher than those reported in the literature obtained from pyro and sol-gel (14 and 3 m^2^ g^−1^) [31] routes as well as from hydrothermal (59 m^2^ g^−1^) [34] and sono-chemical (59 m^2^ g^−1^) [35] methods.

**Figure 4:**
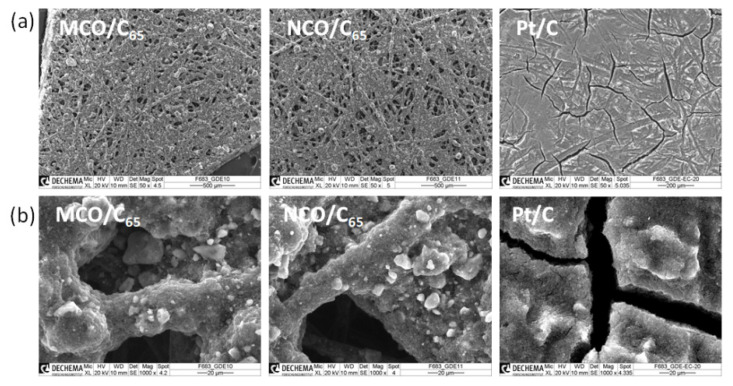
SEM images of different 1 mg_catalyst_ g^−^^1^ GDE surfaces at (**a**) 50× and (**b**) 1000× magnification.

Figure 4 shows SEM images of the spinel GDE surface structures fabricated by the spray coating method. All samples contain an identical catalyst loading of 1 mg cm^−2^ that was mixed with 20 wt.% carbon black C_65_ and 20 wt.% PTFE binder. For comparison, a commercial Pt GDE with identical composition was considered (Figure 4). While the carbon fiber network from the Toray paper structure is still visible on MCO and NCO spinel GDEs, the gas diffusion layer (GDL) surface of Pt GDE is, with exception of crevasse regions, completely covered by the reaction layer. This is principally due to the smaller size and lower density of Pt particles compared to spinel systems that were mixed with a smaller amount of carbon particles and consequently led to a thinner reaction layer. To some extent, the difference in density of carbon materials may also play a role. However, information about density of the commercial carbon product as well as GDL manufacturer is not mentioned on its data sheet. The cracks visible in the commercial Pt/C reaction layer are typical for 1 mg_Pt_ cm^−2^ loaded GDE.

**Figure 5:**
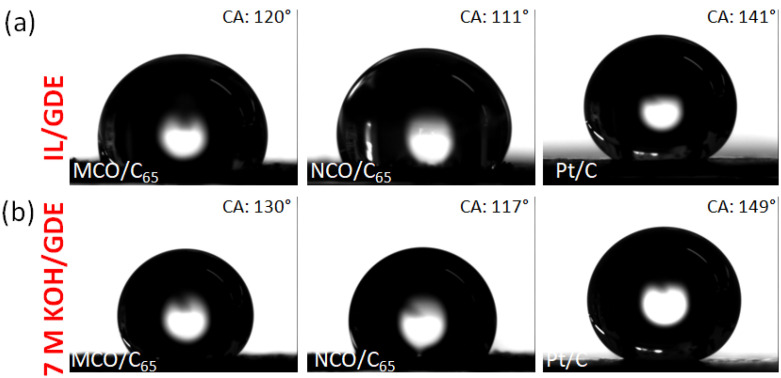
Photos of contact angle (CA) of 5 µL droplets of (**a**) ChAcO + 30 wt.% H_2_O + 0.01 M ZnAcO and (**b**) 7 M KOH after 30 min on different GDE substrates.

The contact angle measurements were carried out in order to investigate the wetting behaviour of choline acetate and KOH on different GDE structures that strongly depends on the surface tension of both liquid and solid phase [36] as well as GDE morphology, porosity, and hydrophobicity. Figure 5 shows the shape of KOH and water-containing ChAcO droplets after 30 min on Pt/C and spinel/C_65_ electrode surfaces. While both KOH and IL droplets form a contact angle higher than 140° with a Pt/C surface, some discrepancies in terms of droplet form and contact angle value are visible on spinel materials. It appears that MCO-based GDE is a-priori more adequate for forming slightly higher contact angle values. However, large pores of about 10–20 µm in spinel GDL in comparison to a maximum of 5 µm in case of commercial GDE observed on TEM images appears to be a handicap. Some additional experiments (not shown here) were performed with choline acetate for up to 100 h in which no substantial change in droplet shape and angles was observed. This was a promising prerequisite for forming a triple-phase boundary [37].

**Figure 6:**
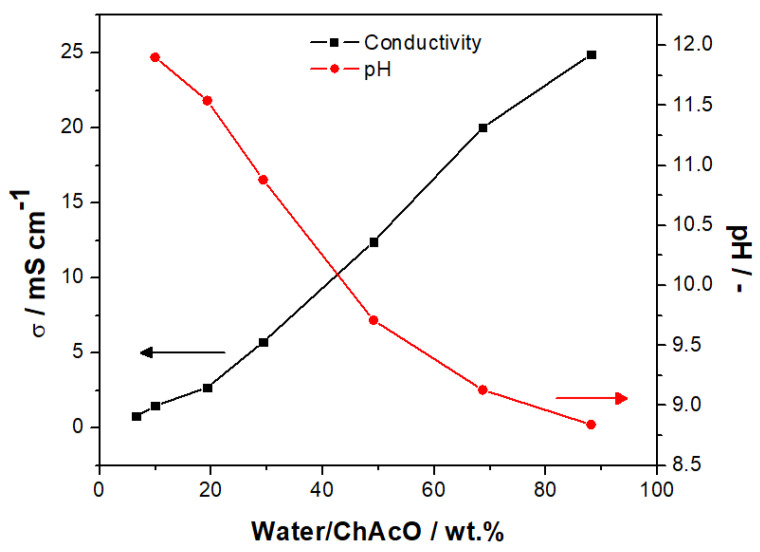
Ionic conductivity and pH values in dependency of water/ChAcO mass ratio at 20 °C. The corresponding conductivity and pH as well as density values are listed in Table S1.

Choline acetate is a highly hygroscopic salt that forms with an appropriate amount of water and an aqueous ionic liquid even in humid air. Below 10 wt.% water, however, the mixture is rather a milky melt with high viscosity and low ionic conductivity. In the range of 20–70 wt.%, a linear correlation between water content and conductivity can be seen in Figure 6, which culminates at 24.9 mS cm^−1^ for the 88% water to a ChAcO mass ratio (47% water to electrolyte mass ratio).

The dependence of pH on water content shows a reverse trend. For 10 wt.% ratio, a pH value close to 12 was measured. Between 20 and 50 wt.%, a linear decline of pH can be observed, whereupon a plateau evolves in proximity to a pH of 8. Thus, the aqueous IL electrolyte exhibits alkaline behavior within a broad range of water/ChAcO ratios. The conductivity and pH value of the 30 wt.% mixture was determined by slight extrapolation to 5.9 mS cm^−1^ and 10.8, respectively.

### 3.2. Half-Cell Investigations

#### 3.2.1. Activity of MCO and NCO Spinel GDEs for ORR/OER in 7 M KOH with Synthetic Dry Air

**Figure 7:**
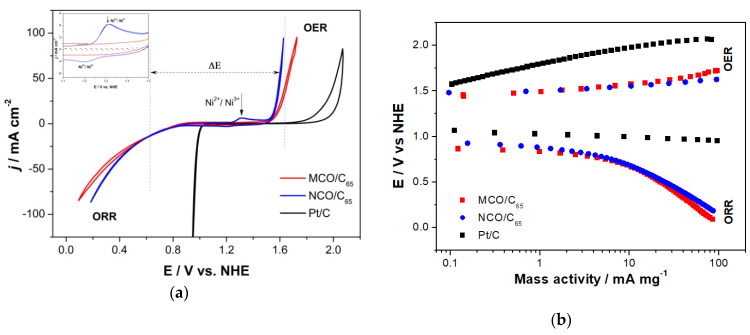
(**a**) CV of 1 mg_catalyst_ cm^−2^ as-prepared MCO and NCO based GDEs and commercial Pt/C GDE at 5 mV s^−1^ in O_2_-saturated 7 M KOH. (**b**) Corresponding semi-logarithmic Tafel plots in ORR/OER regions.

The activity of different spinel for ORR/OER reactions was investigated first in 7 M KOH alkaline electrolyte by cyclic voltammogram (CV) and compared with that of Pt (see Figure 7). While spinel catalysts exhibited similar activity for OER reactions that are at least about 300 mV lower than that observed at Pt at 50 mA cm^−2^, an inverse trend is observed during ORR. At the same current density, a huge potential shift of about to 600 mV to more negative values occurred with spinel GDEs when compared to the Pt one. Since particle size amounts to about 10 nm for commercial Pt and 10–100 nm for as-prepared NCO, a definitive conclusion about the comparative intrinsic activity of both systems is not straightforward. Emerging of additional peaks in voltammogram of Ni-based spinel within the potential range between 1.2 and 1.4 V (inlet in Figure 7a) is attributed to Ni^2+^/Ni^3+^ redox couple and an indication for Ni segregation from the NCO spinel structure. A similar phenomenon was observed at LaNiO_3_ perovskite and is reported in reference [38].

In Figure 7b, potential/current behaviour of MCO/C_65_ and NCO/C_65_ containing GDEs is plotted in function of mass-normalized current density for OER and ORR reactions in a semi-logarithmic diagram and compared to that of Pt/C. In the Tafel plot of spinel systems, one can distinguish two main domains with a transition region close to 10 mA cm^−^^2^ for both OER and ORR reactions. All spinel samples exhibit very similar characteristics.

The Tafel slopes are listed in Table 2 where the first value is assigned to 1 to 10 and second one from 10 to 100 mA cm^−^^2^ current density regions, respectively. These results are in good agreement with Wang et al. [39] work in which binder-free bifunctional NiCo_2_O_4_@NiO and NiFe/NiCo_2_O_4_/NF were tested in 1.0 M KOH electrolyte [40]. Most relevant data related to the activity of different catalysts for ORR/OER such as mass activity (MA), Tafel slope, and *ΔE* are summarized in Table 2.

#### 3.2.2. Activity of MCO for OER/ORR in ChAcO and KOH in Dry Air

**Figure 8:**
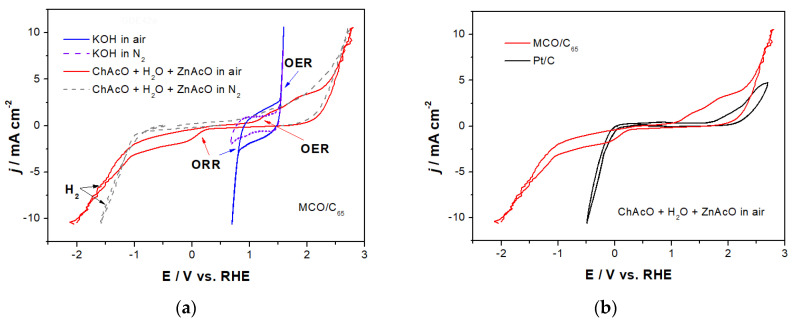
(**a**) OER/ORR activity of MCO in ChAcO + 30 wt.% H_2_O electrolyte at 10 mV s^−1^ with dry synthetic air. CVs in 7 M KOH electrolyte are shown for comparison. (**b**) Overlay of OER/ORR measurements at MCO and Pt in ChAcO + 30 wt.% H_2_O + 0.01 M ZnAcO.

Since half-cell measurements of spinel in oxygen-saturated choline acetate were not easy to interpret because of the apparition of numerous peaks and NCO catalyst was not stable enough, we decided to show only those related to the MCO system for better clarity. Figure 8a shows activity of an MCO gas diffusion electrode (GDE) for oxygen evolution (OER) and reduction (ORR) in water-containing choline acetate with and without zinc acetate salt.

The onset potential of ORR reaction at MCO was evaluated from the difference in charge and peak shape between voltammograms recorded in air-saturated electrolyte and those in nitrogen and, accordingly, located at about +200 and +750 mV for CV in ChAcO and KOH electrolyte, respectively. The ORR reaction is strongly mass-transport limited once reaching current density close to 2.5 mA cm^−^^2^ value. The OER reaction kinetics relies on OH^−^ species’ concentration and transport in electrolyte as well and arises at about +1200 mV in ChAcO and +1500 mV in KOH solution. Thus, a difference in onset potential between OER and ORR (ΔE) in ChAcO amounts to 1 V compared to 750 mV in KOH. By further comparison of MCO onset potentials in water-containing ChAcO with those of Pt (ΔE = 1.4 V) in Figure 8b, it appears that the spinel catalyst is more efficient. The onset potential (OP) values of different MCO and Pt catalyst for OER/ORR as well as corresponding ΔE values are listed in Table 3.

The high increase in current density of up to 10 mA cm^−2^ observed in both vertex potential regions can be associated to superposition of O_2_ evolution with ChAcO oxidation in the anodic scan and H_2_ evolution with cathodic ChAcO decomposition in a cathodic scan. Apparently, the presence of 0.01 M ZnAcO in choline acetate does not significantly affect activity of MCO for ORR/OER reactions.

#### 3.2.3. Long-Term Activity of MCO for OER/ORR in ChAcO in Dry Air

**Figure 9:**
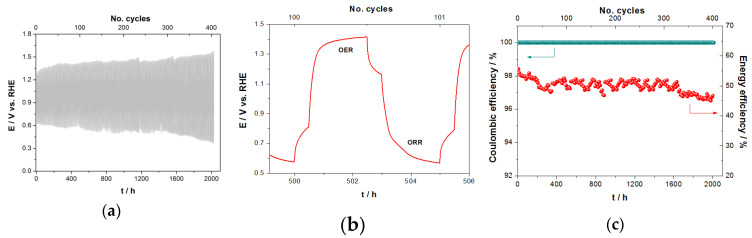
(**a**) Stationary current polarization characteristics of MCO GDE for ORR/OER as a function of time in ChAcO + 30 wt.% H_2_O electrolyte at 100 µA cm^−^^2^ with dry synthetic air. (**b**) 100th cycle and (**c**) corresponding energy and coulombic efficiency.

Electrochemical stability of as-prepared MCO/C_65_ GDE for OER and ORR reactions was alternately tested for 2 h in ChAcO + 30 wt.% H_2_O + 0.01 M ZnAcO with synthetic air at room temperature. At constant polarization value of 100 µA cm^−2^, the GDE was able to perform about 2000 h of operation with 100% coulombic efficiency (see Figure 9a). MCO/C_65_ GDE potential remained stable over 1400 h (280 cycles) with an energy efficiency of 52% when whole charge during ORR and OER steps is considered. At higher cycle number, however, overpotential of MCO for both OER and ORR reactions continuously increased and decreased, respectively, which confirms catalyst degradation as reported elsewhere [41]. This is, to our knowledge, the best result reported in the literature so far. However, as can been observed in a single cycle (Figure 9b), it took a while to reach steady-state values of 1.4 and 0.6 V for OER and ORR, respectively, which is a clear indication for a transport limitation. Moreover, the potential gap after 30 min OCV between OER and ORR steps amounts up to 400 mV that emphasizes sluggish transition from both OER and ORR mode to an equilibrium state.

#### 3.2.4. Activity of Redox Zinc Reactions in ChAcO and KOH Electrolytes

**Figure 10:**
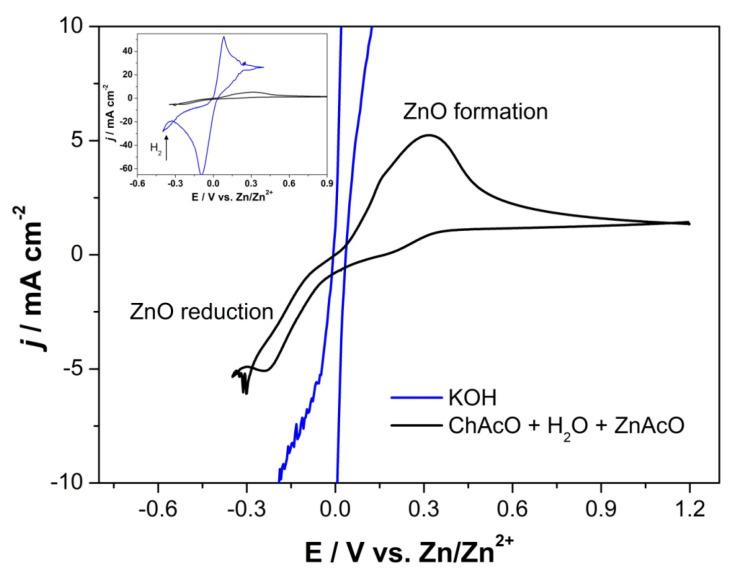
CV of a zinc foil in (black) 30 wt.% water-containing ChAcO + 0.01 M ZnAcO and (blue) 7 M KOH electrolyte at 10 mV s^−1^. Inlet: zoom out of Zn oxidation/ZnO reduction voltammogram in 7 M KOH.

Similarly, activity of zinc oxidation and zinc oxide reduction was investigated in 30 wt.% water-containing choline acetate and compared with that in 7 M KOH (see Figure 10). Due to the relatively strong alkaline nature of aqueous choline acetate (pH~11), we assume that, first, zinc dissolves into zincate that further precipitates into zinc oxide on zinc foil after reaching saturation at an electrode/electrolyte interface similarly to the reaction mechanisms in 7 M KOH. ZnO formation is supported by the Pourbaix diagram of 0.01 M zinc in aqueous medium shown in Figure S4. This assumption will be confirmed by Raman investigation reported in Section 3.4. Sluggishness of zinc reactions in aqueous ionic liquid electrolyte and especially that of zinc oxide reduction is clear. The reaction kinetics depends on available “free” water molecules. In Liu’s work, addition of 10–50 wt.% H_2_O led to substantial enhancement of Zn deposition/stripping activity on a gold substrate from a [EMim]TfO + 0.2 M Zn(TfO)_2_ mixture [42]. In the present study, addition of ZnAcO did not enhance zinc reaction kinetics (not shown here). It clearly appears that not only reaction kinetics of both zinc reactions are faster in 7 M KOH, but also potential gap between both oxidation and reduction peak maxima is considerably narrower in KOH (170 mV) than that in choline acetate (500 mV). Moreover, peak intensity of zinc reactions in aqueous choline acetate is about 10 times lower than those in 7 M KOH. This should be related to the low concentration of available hydroxide ions as well as poor ionic conductivity of choline acetate solution by an approximate factor of 100 compared to that of the 7 M KOH solution.

### 3.3. Zn/air Full-Cell Measurements

#### 3.3.1. Zn/Air U/I Tests in ChAcO and KOH with Synthetic Dry Air in El-Cell

Tests under dry air condition in El-Cell laboratory cells addressed the determination of the intrinsic activity of zinc/air compounds in 30 wt.% water-containing IL electrolyte when compared to that in KOH solution. However, used El-Cell design was not really adapted for long-term tests with the KOH electrolyte. Therefore, a fast charge/discharge procedure of one hour per cycle was applied in order to reduce the experiment’s duration and minimize diffusion and evaporation of alkaline solution. A Pt/C-based GDE served as an air reference electrode in this work. In the present U/I test protocol, maximal reversible capacity yielded per cycle was about 0.063 mAh at 100 µA cm^−2^. This represents about 0.1% depth of discharge (DOD) of the 73.8 mAh theoretical value by assuming a complete dissolution of the zinc foil (90 mg) and neglecting the contribution of dissolved 0.01 M ZnAcO. The photographs of the Zn anodes before and after operation in 7 M KOH and choline acetate are shown in Figure S5.

**Figure 11:**
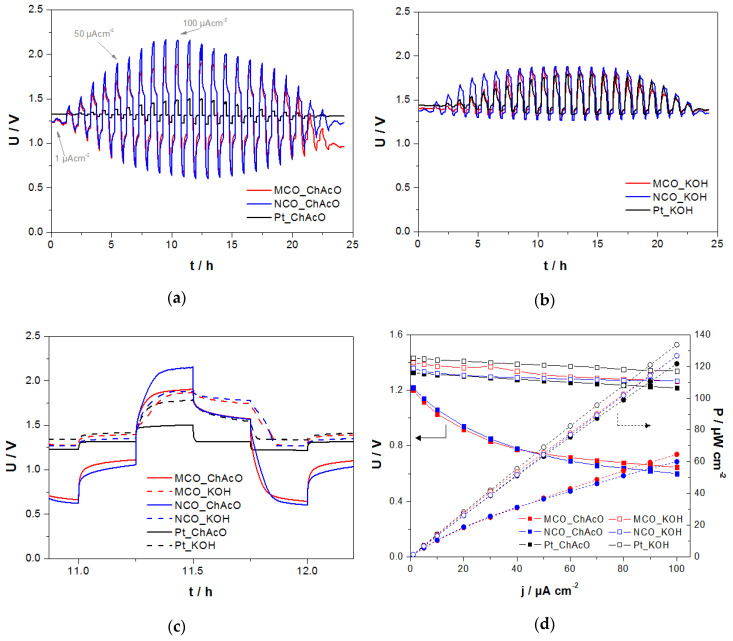
U/I curves of different Zn/air cells between 1 and 100 µA cm^−2^ for 1 h per cycle (15 min charge/discharge each) with dry air in (**a**) 30 wt.% ChAcO + 0.01 M ZnAcO and (**b**) 7 M KOH solutions. (**c**) Selection of a single cycle at 100 µA cm^−2^ in (solid lines) choline acetate and (dotted lines) KOH. (**d**) Comparison of polarization (solid lines) and power density (dotted lines) behavior during the discharge protocol in ChAoC from Figure 11a and KOH from Figure 11b at current increment from 1 to 100 µA cm^−2^ in the forward direction.

In Figure 11, cells with Pt GDE exhibits the highest OCV value in both electrolytes of about 1.33 V in ChAcO and 1.44 V in KOH. All the OCV values are listed in Table S2. While in KOH electrolyte, all cells showed a comparable charge and discharge profiles, cells with MCO or NCO gas diffusion electrode exhibited poor activity for both charge and discharge steps in choline acetate. Best overall cell performance was yielded with a Pt-equipped cell in aqueous choline acetate whereas an inverse trend was observed in the cell with Pt in KOH. In that case, overpotential values were clearly lower than those in the KOH solution. Figure 11d shows the polarization and power density curves of the different zn/air cells during the discharge step in both ChAcO and KOH electrolyte with dry air. While maximum peak power density of Zn/air cell with Pt/C amounts to about 122 µW cm^−2^ at 100 mA cm^−2^ and is very close to the values of all Zn/air cells in KOH (125–135 µW cm^−2^), Zn/air cells with spinel catalysts exhibit about 50% lower performance in an aqueous IL electrolyte.

#### 3.3.2. Zn/Air I-U Tests in ChAcO with Ambient Air in El-Cell

**Figure 12:**
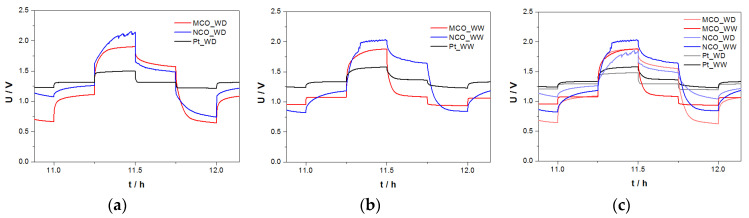
Comparison of 1-h charge/discharge profiles of different Zn/air cells at 100 µA cm^−2^ during U/I test procedure in (**a**) 30 wt.% water-containing choline acetate electrolyte—dry air (WD) and (**b**) 30 wt.% water-containing choline acetate electrolyte—ambient air feed (30% RH at 23°C) (WW). (**c**) Overlay of Figure 12a,b.

Similar protocol as in Section 3.3.1 was used for the U/I experiment in ambient air. For better clarity, however, only cycles carried out at 100 µA cm^−^^2^ (2.8 mA g^−^^1^_zinc_) are shown in Figure 12a–c where evidence effects the moisture in air on short charge/discharge (15 min each) polarization time of different Zn/air cells.

The following conclusions can be drawn: (i) Charge/discharge characteristics of cell with Pt as a catalyst air electrode surpassed those with MCO and NCO by far, (ii) best performances with Pt cells were yielded with dry air, it appears that the presence of humidity in air affected kinetics of Pt for both charge (OER) and discharge (ORR) reaction step, (iii) performance of cell with NCO decayed under dry air feed, and (iv) substantially positive effect of moisture in air feed on discharge (ORR) step of cell with MCO while cell voltage profiles during charging steps remained almost unchanged and were apparently insensitive to water.

In this preliminary survey, cells with the NCO air electrode demonstrated poor cycling ability, especially during charging steps. Therefore, this catalyst was not considered in further investigations.

#### 3.3.3. Zn/Air Charge/Discharge Test in ChAcO for 26 h/Cycle in the El-Cell

**Figure 13:**
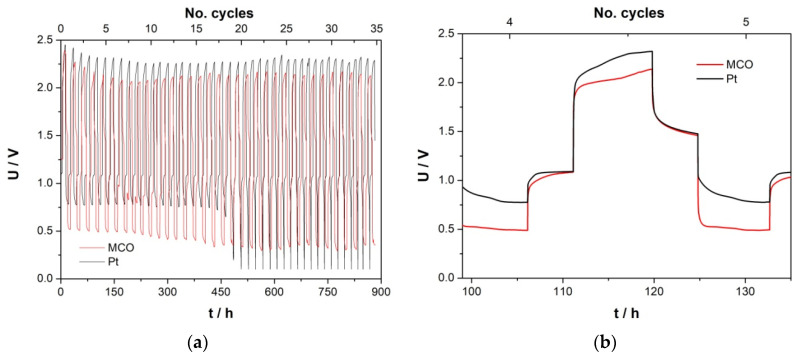
(**a**) Average values of zinc/air cell performance in 30 wt.% H_2_O/ChAcO + 0.01 M ZnAcO_2_ electrolyte during cycling at 100 µA cm^−2^ (2.82 mA g^−1^_zinc_) in ambient air for 26 h per cycle (8 h charge/discharge each) in El-Cell. (**b**) Representation of the fifth cycle. Voltage cut-off limits were fixed to 2.5 and 0.1 V. The absolute charge and discharge capacity value was limited to 2.2 and 2.0 mAh, respectively.

For the majority of battery applications, either high-power density for short duration or high capacity over a long period in ambient air is required. Since zinc/air battery with choline acetate ionic liquid electrolyte can clearly not deliver high power densities, the next experiments shown in Figure 13 focus on an extension of cycle duration up to 8 h of the charge/discharge step (5 h OCV)) at 100 µA cm^−^^2^ (2.8 mA g^−^^1^_zinc_) with either Pt or MCO as a bifunctional air catalyst in ambient air.

One can divide 26 h/cycle experiment depicted in Figure 13a into two main regions. Within the first 15 cycles, cells with a Pt electrode clearly over-performed those with an MCO GDE, especially during the discharge step. The reason why the mean discharge potential of the Pt cells suddenly dropped from 0.75 V down to 0.15 V is not explicable yet. Cells with an MCO catalyst were relatively stable over 34 days.

Figure 13b shows overlay of the fifth cycle of cells that can be commented as follows. (i) Even after 8 h of charge, no single cell reached steady-state potential. (ii) In case of the discharge step, achievement of steady-state is faster in the MCO cell than in the Pt one. (iii) OCV of both systems tended to approach 1 V value. (iv) Superior performance of Pt cell during discharge and of MCO cells during charge is in good accordance with ORR/OER half-cell measurements. (v) Clear discrepancy with respect to charging voltage of cell with a Pt catalyst in comparison with short U/I polarization time (2.2 V in average vs. 1.5 V in Figure 11b). No explanation for this behaviour has been found yet.

#### 3.3.4. Zinc/Air Charge/Discharge Tests in ChAcO for 10 Days/Cycle in the EL-Cell

**Figure 14:**
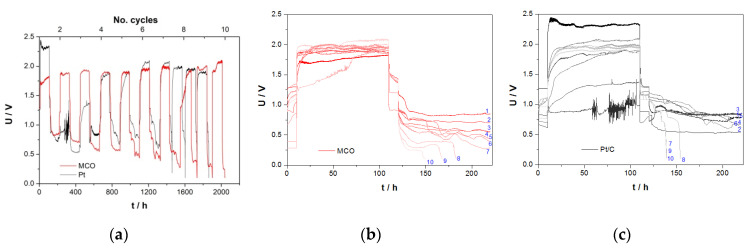
(**a**) Long-term stability cycling test of MCO and Pt cells in ChAcO + 0.01 M ZnAcO_2_ + 30 wt.% H_2_O cell at 100 µA cm^−2^ for about 10 days/cycle in ambient air (30% RH at 23 °C). (**b**,**c**) Comparison of all charge/discharge steps of corresponding GDE. Cut-off limits 2.5 and 0.1 V.

The best performing MCO and commercial Pt/C GDEs were further tested for a very large period of 100 h for a charge and discharge step each (about 10 days per cycle) in Zn/air cell under ambient air conditions (Figure 14). Even though polarization curves are not very flat, Zn/air cell with MCO air electrode exhibited clearly better performance than the cell with Pt GDE.

Moreover, reversible capacity of zinc/air battery amounted to 25.4 mAh that corresponds to about 28% DOD at 100% columbic efficiency. This excellent behaviour was observed until the first 7th (1500 h) and 6th (1300 h) cycles for MCO and Pt cells, respectively.

#### 3.3.5. Zn/Air Single Discharge Step in ChAcO for 12 h/Cycle in El-Cell

Next, the investigation aimed at determining maximum cell discharge capacity of a pristine zinc foil at 100 μA cm^−2^ under ambient air condition.

**Figure 15:**
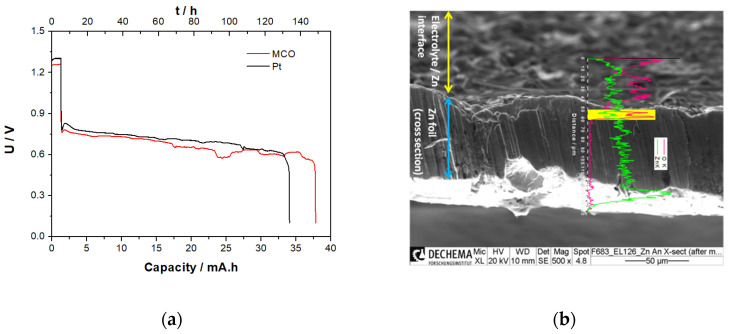
(**a**) Discharge profile of zinc/air cell with MCO and Pt as GDE at 100 µA cm^−2^ and (**b**) SEM image of a cross-sectional Zn electrode after compete discharge of ChAcO + 0.01 M ZnAcO_2_ + 30% H_2_O cell containing MCO GDE at 100 µA cm^−2^ in ambient air with an EDX signal of specific elemental count rate profile in line scan for oxygen (red) and zinc (green) along and zinc surface in contact with an electrolyte, zinc foil cross section, and zinc foil in contact with the current collector (bright area). An enlarged SEM and line scan image are provided in Figure S6 for better readability.

In 30% RH air atmosphere, capacities of Zn/air battery using Pt/C and MCO/C as GDE amounted to 34.3 and 38.1 mAh that represents 38% and 42% DOD values, respectively (see polarization profile in Figure 15a). After these discharge tests, however, it was not possible to recharge the cells again, presumably due to irreversible formation of dense zinc oxide/hydroxide layer and irreversible passivation of the electrode surface. This is confirmed by SEM-EDX elemental mapping and line scan of post-mortem analysis of zinc foil shown in Figure 14b where evidence of high oxygen concentration on the zinc surface and near-surface layer in a depth of about 10 µm (yellow-marked area in Figure 15b).

#### 3.3.6. Zn/Air Charge/Discharge Steps for 12 h/Cycle in CR2032 Coin Cell

**Figure 16:**
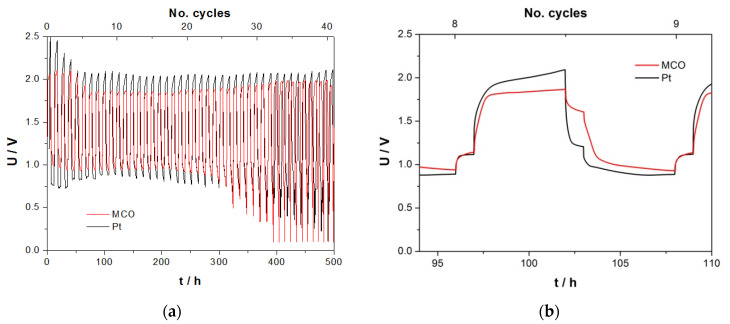
(**a**) Long-term stability cycle tests in zinc/air coin cell with MCO or Pt as an air electrode in ChAcO + 0.01 M ZnAcO_2_ + 30% H_2_O electrolyte for 12 h per cycle (5 h charge/discharge) at 100 µAcm^−2^ in ambient air (30% RH at 23°C). (**b**) Comparison of charge/discharge profile during 18th cycle. Voltage cut-off limits of 2.5 and 0.1 V.

Figure 16 illustrates the behaviour of zinc/air cell with either MCO or Pt as an air catalyst in aqueous choline acetate for 5-h charge/discharge step in coin cell design. The reversible capacity was 1.01 mAh (1.4% DOD). Although current density amounted to the same value of 100 µA cm^−2^ (2.80 mA g^−1^_zinc_), steady-state operation seems to be reached at 1.80 and 0.94 V for the charge and discharge step, repectively, in case of the cell with an MCO air electrode, which represents an energy efficiency of 52% for about 300 h.

The CR2032 coin cell design with 16 mm in diameter zinc foil appears to be more adapted for long-term experiments than that of El-Cell. Some relevant OCV values are listed in Table S2.

Table 4 summarized all results from Zn/air charge/discharge experiments of this work. Best performance in terms of test duration, reversible capacity, and energy efficiency were yielded in El-cell design with an MCO air catalyst during a 100 h charge/discharge protocol.

### 3.4. In-Situ Raman Investigation in the Side-by-Side Zn/Air Cell

**Figure 17:**
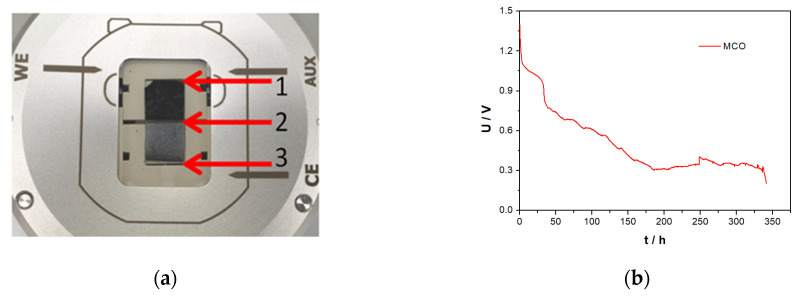
(**a**) SBS cell design (1:GDE, 2:separator, 3:Zn/carbon paper) and (**b**) Zn/air cell discharge profile in ChAcO + 30 wt.% H_2_O at 5 µA cm^−2^.

In order to identify reaction products during zinc/air cell discharge process in 30 wt.% water-containing choline acetate, an *in-situ* Raman spectroscopy was carried out at different DODs in an optical SBS cell displayed in Figure 17.

**Figure 18:**
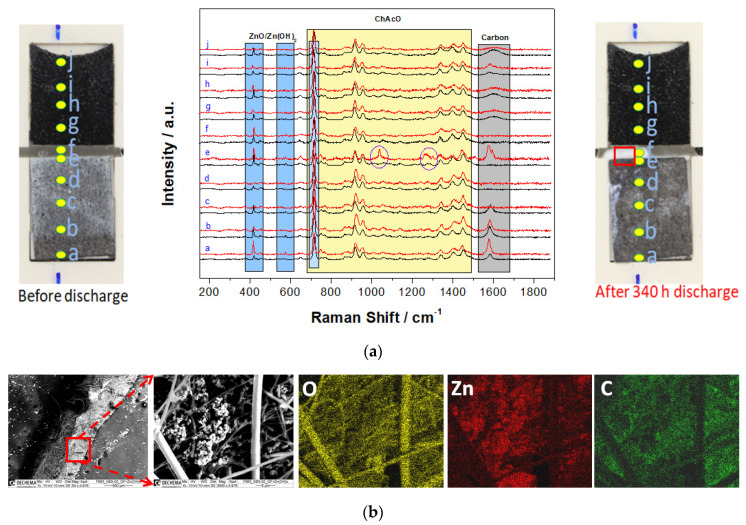
(**a**) Raman line scanning spectra at 10 identical positions of electrodes, separators, and electrolytes with a photo of system set-up (black) before and (red) after the discharge procedure. (**b**) SEM and EDX mapping images of discharge product in glass fiber separator in a red marked square region.

For this special cell design, however, it was necessary to use highly porous zinc particles for appropriated wetting. Accordingly, a zinc layer was previously electrodeposited on a carbon paper substrate. A line-scan consisting of 10 Raman spectra was performed at equidistant locations every 10% DOD. The cell delivered an initial OCV of 1.4 V and a constant current discharge step was carried out at 5 µA cm^−^^2^ for 340 h that represents a capacity of 1.75 mAh (~9.4% DOD). For higher clarity, however, only spectra before and after complete discharge are shown in Figure 18a. The formation of the reaction product in a form of white precipitate layer was observed at the zinc anode and glass fibre separator interface, as can be seen in a red window displayed in Figure 18a. However, the GDE surface was nearly unchanged.

Figure 18a shows superposition of Raman spectra of the Zn/air cell surface at 10 identical locations (a-j position) before and after cell discharge. After 340 h of discharge, two strong bands at ca. 1040 and 1270 cm^−^^1^ within the electrolyte separator (position e in Figure 18a) indicate zincate formation [43]. Two other strong bands at 720 and 1603 cm^−^^1^ are assigned to OH rotational modes [44]. The presence of the zinc oxide compound is also shown by SEM-EDX mapping, as shown in Figure 18b. The peak at 1583 cm^−^^1^ (G peak) in Raman spectra is assigned to sp^2^ hybridized carbon from Toray carbon fibers.

## 4. Conclusions

In this work, feasibility of a zinc/air cell operating with a 30 wt.% H_2_O-containing choline acetate as electrolyte and MnCo_2_O_4_ as a bifunctional air electrode at 100 µA cm^−2^ for up to 100 h charge/discharge step for about 1500 h under ambient air condition was successfully demonstrated in El-Cell. Best results in terms of energy efficiency up to 52% for 300 h (25 cycles) were obtained during a 5 h charge/discharge step in the CR2032 coin cell. Due to a relatively high pH value of about 11, reaction products such as zinc oxide/hydroxide of zinc/air cell with aqueous ionic liquid were as expected, which is similar to those with highly concentrated potassium hydroxide. In contrast to experiments in KOH, neither zinc dendrite nor carbonate formation was detected in choline acetate-based electrolyte. The use of a zinc foil as a negative electrode was principally motivated by its facile integration into El-Cell and CR2032 coin cell designs. Relatively high reversible capacities up to 25.4 mAh (28% DOD) were yielded despite a smooth and dense surface of zinc foil material. Further work aims to improve the electrode design and cell configuration in order to achieve higher reversible capacity and current density values by using zinc powder and a thinner separator for rechargeable zinc/air applications. The amount of cobalt in the bifunctional air electrode has to be reduced as well.

## Figures and Tables

**Table 1:** Physical properties of bifunctional catalysts.

Catalyst	BET m^2^ g^−1^	Pore Size ^+^ nm	D ^#^ nm	CA * °	CA ** °
MCO/C_65_	126	17	26	120	130
NCO/C_65_	108	3.8	100	111	117
Pt/C	55	36	10	141	149

^+^ average pore diameter, ^#^ average particle diameter, * contact angle of IL on GDE surface measured at room temperature (RT) for 30 min, ** contact angle of 7 M KOH on GDE surface measured at RT for 30 min. All contact angle (CA) values are given with an error bar of ±1°.

**Table 2:** Summary of GDE electrochemical properties from experiments shown in Figure 7.

Catalyst	MA_ORR_ at 0.6 V mA mg^−2^	MA_OER_ at 1.65 V mA mg^−2^	Tafel_ORR_ mV dec^−1^	Tafel_OER_ mV dec^−1^	ΔE at 10 mA cm^−2^ V
MCO/C_65_	15	47	70–185 ^#^	77–113 ^#^	0.9
NCO/C_65_	16	74	84–186 ^#^	30–70 ^#^	0.86
Pt/C	98 *	1.5	60–150 ^+^	178–330 ^+^	0.86

* measured at 0.95 V. ^#^ Tafel slope for ORR and OER was calculated within 0.75–0.9 V and 1.55–1.65 V potential window, respectively. ^+^ Tafel slope for ORR and OER was calculated within 0.95–1.05 V and 1.8–2.0 V potential window, respectively.

**Table 3:** List of onset potential values of different GDEs for OER/ORR in either 7 M KOH or ChAcO + 30 wt.% H_2_O (IL) solution with dry synthetic air from experiments shown in Figure 7 and Figure 8.

GDE Catalyst Electrolyte	MCO IL	MCO KOH	Pt IL	Pt KOH
**OP_OER_/mV ^#^**	1200	1500	1725	1800
**OP_ORR_/mV ^#^**	200	750	35	1000
**ΔE/mV**	1000	750	1690	800

^#^: onset potential (OP) vs. RHE

**Table 4:** Summary of relevant results of Zn/air charge/discharge experiments at 100 µA cm^−2^ (2.8 mA∙g^−1^_zinc_) in ChAcO + 30 wt.% H_2_O + 0.01 M ZnAcO_2_ and ambient air.

Design	El-Cell				Coin Cell	
Current/µA	254				200	
Time per cycle/h	26		240		12	
Rev. capacity/mAh	2.03		25.40		1.01	
DOD/%	2.2		28.0		1.4	
Air catalyst	Pt	MCO	Pt	MCO	Pt	MCO
Cycles number/ *-	1–17	1–33	1–6	1–7	1–30	1–33
Duration/h *	440	850	1300	1500	360	396
Coulombic efficiency/% *	100	100	100	100	100	100
Energy efficiency/%*	28–35	15–24	37–51	29–54	39–48	33–56

* Values related to the region in which relatively stable cell voltage behavior was observed.

## Supplementary Materials

The following are available online at https://www.mdpi.com/1996-1944/13/13/2975/s1. Figure S1: (a) Schematic representation and picture of El-Cell configuration (SS–stainless steel). (b) Picture of coin cell type CR2032 with holes provided for air access at the cathode cap. Electrode arrangement in the coin cell is identical as in El-Cell except the number of separators, which was two 1-mm thick, Figure S2: SEM images and EDX pattern of as-prepared (a) MCO and (b) NCO powder sample, Figure S3: BET isotherm curve of (a) MCO, (b) NCO, and (c) Pt/C. (d–f). Corresponding pore size distribution plots, Figure S4: Pourbaix diagram of 0.01 M zinc in aqueous solution from materials project.org, Figure S5: Photographs of Zn/air El-Cell components (top) before experiments, (middle) after 20 days test in choline acetate, and (bottom) 1-day test in 7 M KOH. (GF = glass fiber separator), Figure S6: SEM image of cross-sectional Zn electrode after compete discharge of ChAcO + 0.01 M ZnAcO_2_ + 30% H_2_O cell containing MCO GDE at 100 µA cm^−2^ in ambient air with an EDX signal of specific elemental count rate profile in line scan for oxygen (red) and zinc (green) along and zinc surface in contact with electrolyte, zinc foil cross section, and zinc foil in contact with the current collector (bright area). Table S1: Physicochemical properties of different water/ChAcO mixtures at 20 °C, Table S2: List of OCV values from different Zn/air cells.
