# Supplementary material for: An Electrically Rechargeable Zinc/Air Cell with an Aqueous Choline Acetate Electrolyte
Source: Materials (Basel). 2020 Jul 3;13(13):2975. doi: 10.3390/ma13132975 (PMC7372399; doi:10.3390/ma13132975)
Supplement: Supplementary file 1 [file materials-13-02975-s001.pdf]

materials

Supplementary Information

An Electrically Rechargeable Zinc/Air Cell with an Aqueous Choline Acetate Electrolyte

Mariappan Sakthivel, Sai Praneet Batchu, Abbas Ali Shah, Kwangmin Kim, Willi Peters and Jean-Francois Drillet *

DECHEMA-Forschungsinstitut, Theodor-Heuss-Allee 25, 60486 Frankfurt am Main, Germany; sakthivel@dechema.de (M.S.); bspraneet@gmail.com (S.P.B.); abbasalishah@live.com (A.A.S.); kwangmin.m.kim@gmail.com (K.K.); peters@dechema.de (W.P.)
* Correspondence: jean.drillet@dechema.de; Tel.: + 49-69-7564-476

Received: 20 May 2020; Accepted: 30 June 2020; Published: date

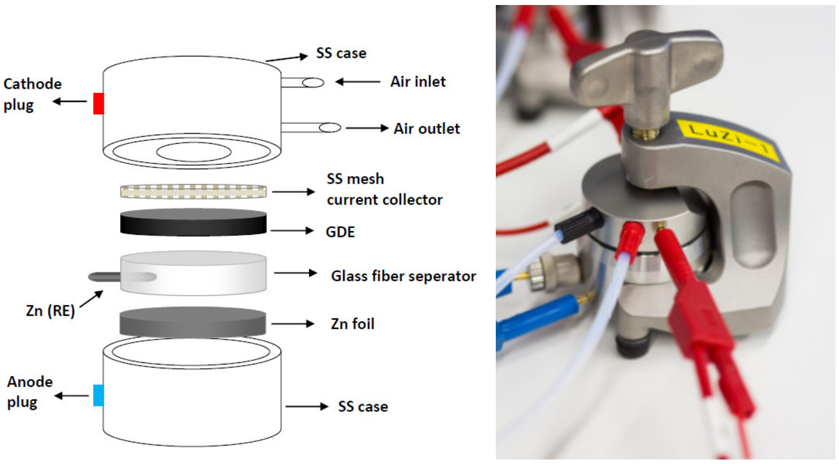

(a)

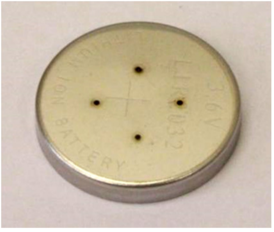

(b)

Figure S1. (**a**) Schematic representation and picture of El-Cell configuration (SS – stainless steel). (**b**) Picture of coin cell type CR2032 with holes provided for air access at cathode cap. Electrode arrangement in coin cell is identical as in El-Cell except the number of separator which was two 1 mm thick.

Figure S2 shows SEM image and EDX pattern of both as-prepared spinel powder catalysts that appear to be very homogeneous in terms of particle size distribution. Moreover no additional impurities from precursor source such as sodium or nitrogen were detected by EDX.

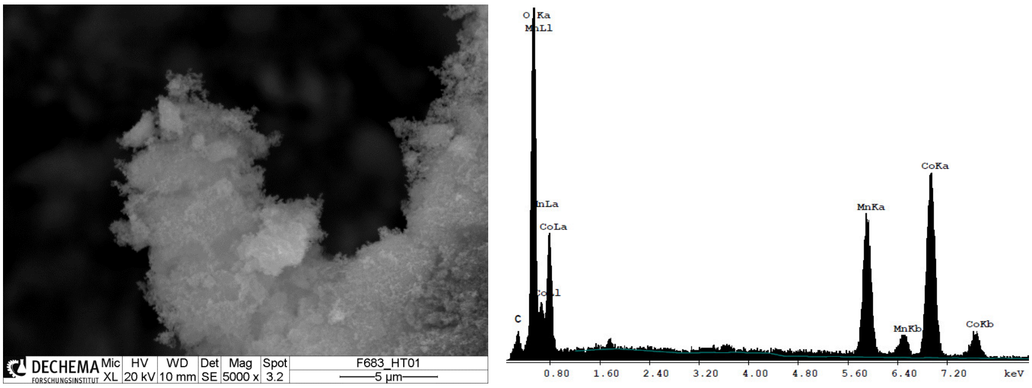

(a)

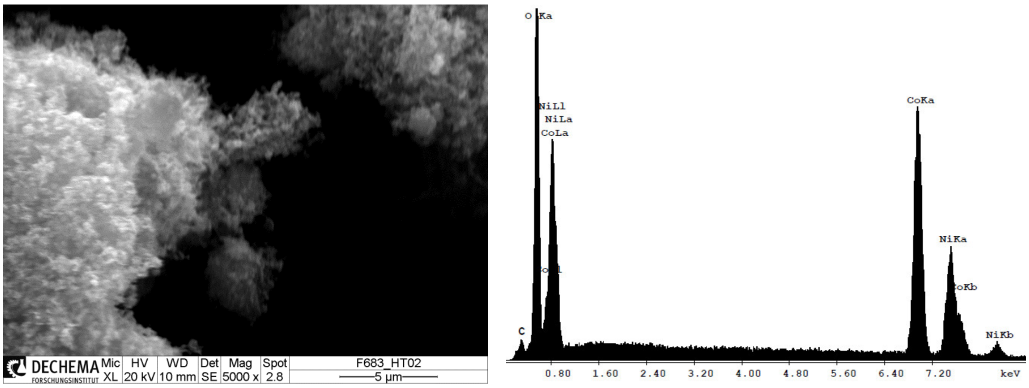

(b)

Figure S2. SEM images and EDX pattern of as-prepared (**a**) MCO and (**b**) NCO powder sample.

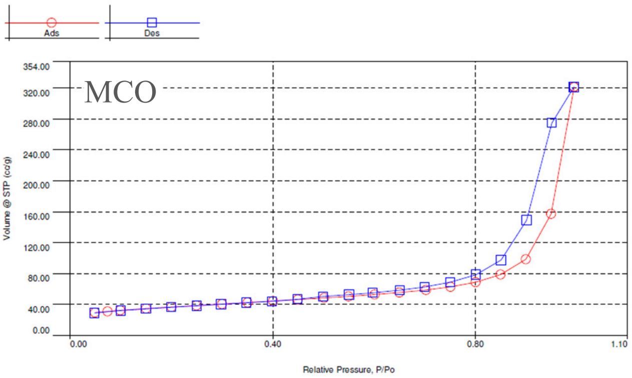

(a)

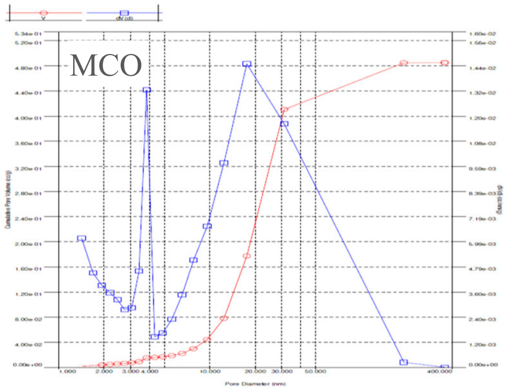

(d)

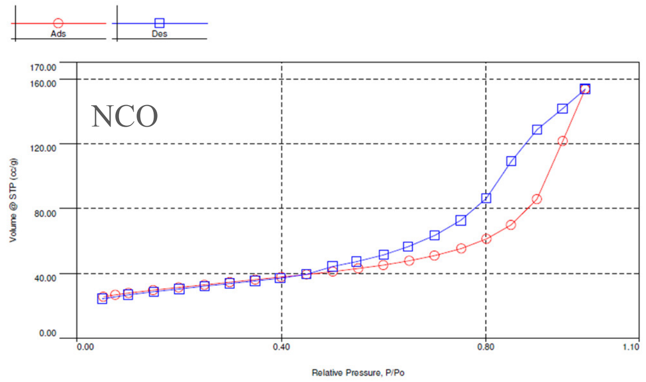

(b)

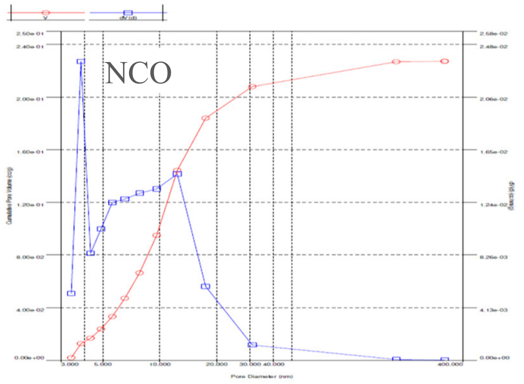

(e)

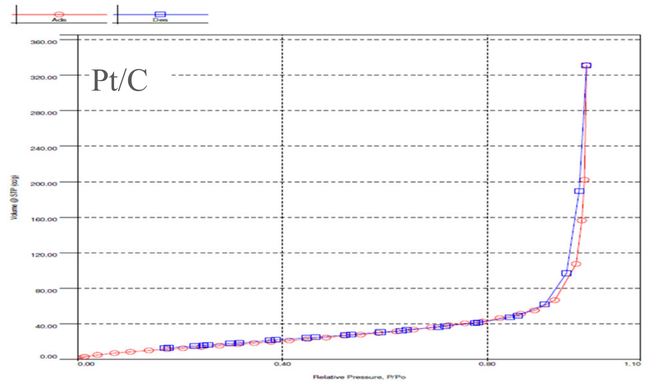

(c)

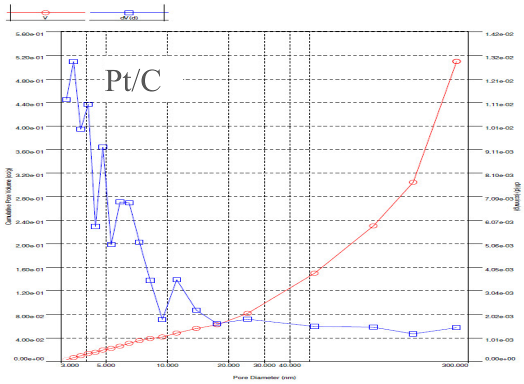

(f)

Figure S3. BET isotherm curve of (**a**) MCO, (**b**) NCO and (**c**) Pt/C. (**d–f**) Corresponding pore size distribution plots.

Table S1. Physicochemical properties of different water/ChAcO mixtures at 20 °C.

	**H_2O/ChAcO Mass Ratio g_{H2O} × g^{-1}_{ChAcO}**	**H_2O Content Mass Ratio g_{H2O} × $g^{-1}_{electrolyte}$**	**H_2O/ChAcO Molar Ratio mol_{H2O} * mol^{-1}_{ChAcO}**	**Conductivity mS cm^{-1}**	**pH -**	**Density g mL^{-1}**
1	6.6%	6.2%	0.61	0.77	-	-
2	10.0%	9.1%	0.92	1.47	11.90	1.094
3	19.3%	16.2%	1.75	2.70	11.54	-
4	29.3%	22.7%	2.66	5.73	10.88	1.090
5	49.1%	32.9%	4.45	12.41	9.71	-
6	68.7%	40.7%	6.22	20.02	9.13	-
7	88.1%	46.8%	7.98	24.90	8.84	1.061

Table S2. List of OCV values from different Zn/air cells.

**Catalyst**	**KOH || Dry Air (V)**	**ChAcO +ZnAcO || Dry Air (V)**	**ChAcO +ZnAcO +H_2O || Dry Air (V)**	**ChAcO +ZnAcO +H_2O || Ambient Air (V)**
MCO/C_{65}	1.4	1.24	1.23	1.21

NCO/C_{65}	1.37	1.25	1.3	1.24
Pt/C	1.44	1.32	1.32	1.33

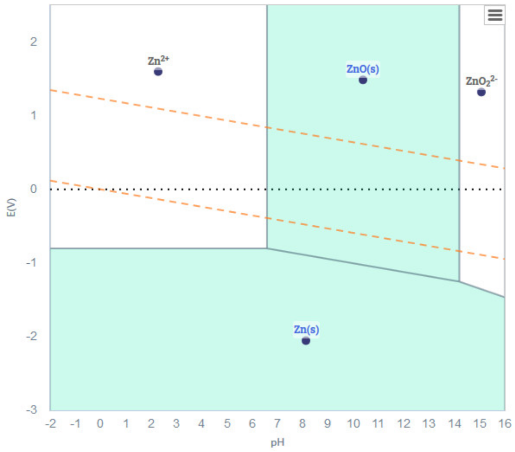

Figure S4. Pourbaix diagram of 0.01 M zinc in aqueous solution from materials project.org [1].

Post-mortem analysis of electrode and separator materials

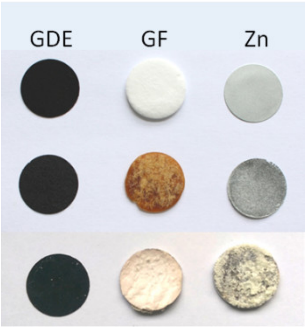

Figure S5. Photographs of Zn/air El-Cell components (top) before experiments, (middle) after 20 days test in choline acetate and (bottom) 1 day test in 7 M KOH. (GF = glass fiber separator).

Figure S5 illustrates the electrode and separator surfaces after operation for one day in 7 M KOH and for twenty days in ChAcO + 0.01 ZnAcO compared to those before the experiments ones. After one day of polarization experiment in 7 M KOH, Zn foil turns into the porous white colored powder, mostly ZnO. This indicates the near complete utilization of zinc in alkaline electrolyte. However in ionic liquid (ChAcO), the zinc foil was almost intact; surprisingly the surface facing the separator which is in contact with electrolyte turned slightly black indicating formation of zinc hydroxide (passivation layer) while few white colored zinc oxide was also observed. The performance of cell in ionic liquid is obviously limited by low capacity of ChAcO to dissolve zinc ions as compared to KOH.

The glass fiber separator was found to be dried after operating with 7 M KOH that is an indication for electrolyte evaporation/migration. In the IL containing cell, trace of electrolyte was still visible on electrodes and separator. In both cases on GDE surface, there were no changes visible to eyes.

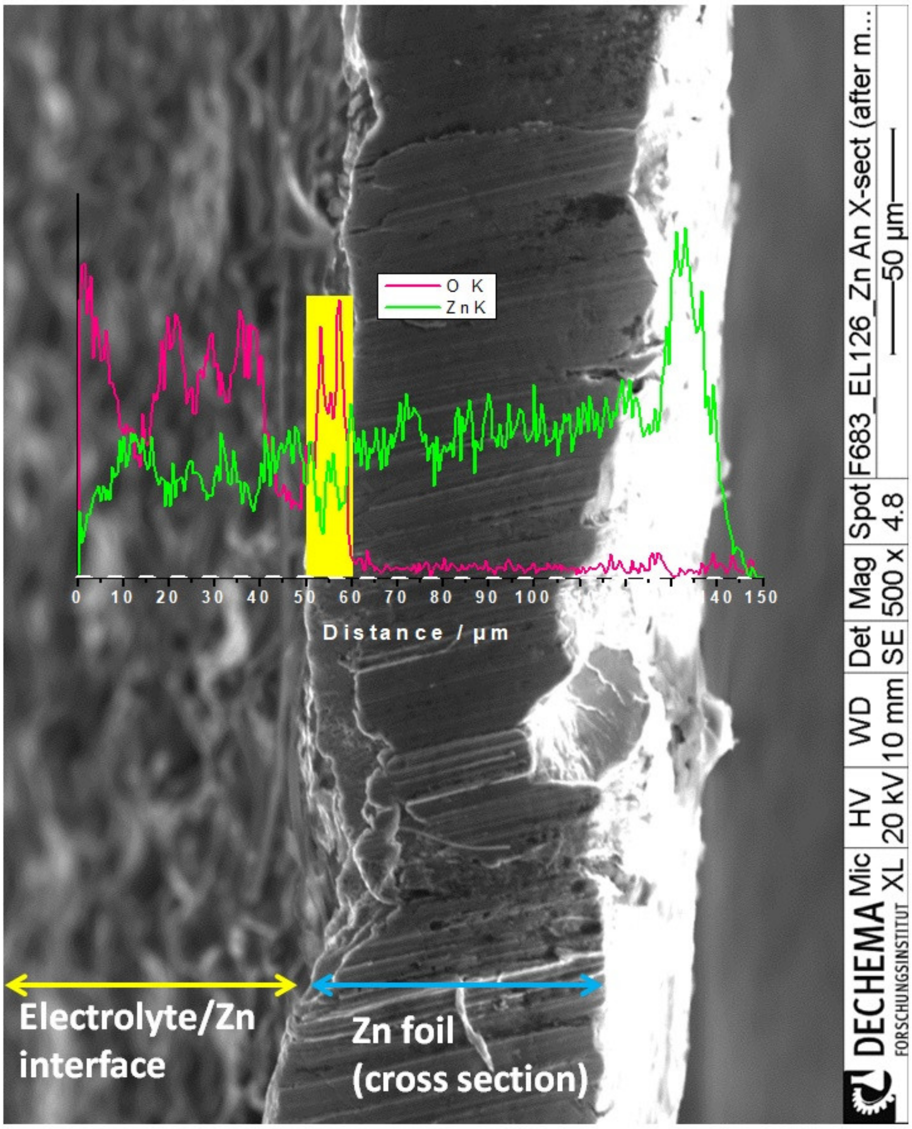

Figure S6. SEM image of cross sectional Zn electrode after compete discharge of ChAcO + 0.01 M $ZnAcO_2$ + 30% H_2O cell containing MCO GDE at 100 µA cm^{-2} in ambient air with EDX signal of specific elemental count rate profile in line scan for oxygen (red) and zinc (green) along and zinc surface in contact with electrolyte, zinc foil cross section and zinc foil in contact with current collector (bright area).

References

1. The Materials Project, www.materialsproject.org (accessed on 19 June 2020).

© 2020 by the authors. Submitted for possible open access publication under the terms and conditions of the Creative Commons Attribution (CC BY) license (http://creativecommons.org/licenses/by/4.0/).
